# Integrative serum proteomic and metabolomic profiling in ovarian endometrioma: an exploratory multi-omics study

**DOI:** 10.3389/fmed.2026.1846827

**Published:** 2026-07-22

**Authors:** Na Chen, Yubing Hu, Tianxia Xiao, Yun Ma, Litong Zhu, Jiahong Gao, Jian V. Zhang, Ping Jin

**Affiliations:** 1 Department of Gynecology, Shenzhen Maternity and Child Healthcare Hospital, Women and Children's Medical Center, Southern Medical University, Shenzhen, Guangdong, China; 2 Center for Energy Metabolism and Reproduction, Shenzhen Institutes of Advanced Technology, Chinese Academy of Sciences, Shenzhen, Guangdong, China; 3 Shenzhen School of Clinical Medicine, Southern Medical University, Shenzhen, Guangdong, China; 4 Shenzhen Key Laboratory of Metabolic Health, Shenzhen Metabolism and Reproductive Targeted Delivery Proof-of-Concept Center, Shenzhen Institutes of Advanced Technology, Chinese Academy of Sciences, Shenzhen, Guangdong, China; 5 Faculty of Pharmaceutical Sciences, Shenzhen University of Advanced Technology, Shenzhen, Guangdong, China; 6 Sino-European Center of Biomedicine and Health, Shenzhen, Guangdong, China

**Keywords:** endometriosis, metabolomics, multi-omics, ovarian endometrioma, proteomics

## Abstract

**Background:**

Ovarian endometrioma (OEM) is a common manifestation of endometriosis and is associated with both local lesions and systemic alterations. While immune dysregulation and metabolic disturbances have been individually reported in endometriosis, whether these changes are coordinated at the circulating molecular level in OEM remains unclear.

**Methods:**

We performed an exploratory integrative analysis of serum proteomic and untargeted metabolomic profiles in women with OEM and healthy controls. Differential analyses were conducted for each omics layer, followed by cross-omics integration to assess pathway-level relationships between molecular alterations. Selected platelet-associated proteins were further evaluated by enzyme-linked immunosorbent assay (ELISA) in a small independent cohort.

**Results:**

Serum proteomics profiling showed predominant alterations in immune-related and extracellular processes, whereas metabolomic analysis revealed changes mainly involving lipid metabolism, amino acid metabolism, and redox-related pathways. Cross-omics integration suggested pathway-level correspondence between the two omics layers, particularly in carbohydrate metabolism, nucleotide metabolism, lipid remodeling, and antioxidant defense. Among platelet-associated proteins, pro-platelet basic protein (PPBP/CXCL7) showed higher levels in OEM in both proteomic profiling and ELISA validation, while MMRN1 and PDGFA showed concordant but non-significant increases in the ELISA cohort.

**Conclusion:**

These findings suggest that OEM is associated with concurrent immune-related and metabolic alterations detectable in the circulation. Integrative multi-omics analysis provides exploratory evidence for pathway-level correspondence between proteomic and metabolomic alterations in OEM and provides limited independent support for a platelet-associated circulating signal, with PPBP showing the most consistent validation.

## 1 Introduction

Endometriosis is a chronic, estrogen-dependent inflammatory disorder characterized by the presence of endometrial-like tissue outside the uterus, affecting approximately 10% of reproductive-aged women (1). Ovarian endometrioma (OEM), commonly referred to as a “chocolate cyst,” represents a major clinical subtype and is frequently associated with pelvic pain, infertility, and an increased risk of endometriosis-related ovarian cancer (2). Although OEM is anatomically confined to the ovary, increasing evidence indicates that its biological effects are not limited to local lesions and may involve broader systemic alterations (3). Despite advances in clinical management, the diagnosis of endometriosis still largely relies on laparoscopic visualization, an invasive procedure that is often associated with substantial diagnostic delay. Imaging techniques can aid in the detection of advanced ovarian lesions but show limited sensitivity for early or atypical disease (1). These challenges highlight the importance of understanding systemic molecular changes associated with OEM, beyond localized pathology.

Previous proteomic studies of endometriosis have reported alterations in immune mediators, extracellular matrix remodeling proteins, complement components, and pathways associated with oxidative stress in both serum and tissue samples (4–7). In parallel, metabolomic studies have identified changes in lipid metabolism, amino acid turnover, and redox homeostasis (8, 9). However, most existing studies have examined proteomic and metabolomic alterations independently. As a result, how immune-related protein changes and metabolic reprogramming coexist or relate to each other at the systemic level in OEM remains insufficiently characterized.

Proteins and metabolites reflect different yet complementary aspects of disease biology. Circulating proteins are often associated with immune activation, signaling processes, and extracellular communication, whereas metabolites represent downstream biochemical adaptations to inflammation and oxidative stress (10, 11). From this perspective, limited overlap between proteomic and metabolomic findings is not unexpected. Integrative multi-omics analysis therefore provides a framework to explore coordinated molecular changes that may not be apparent when each molecular layer is examined in isolation (12). Recent advances in data-independent acquisition mass spectrometry have enabled deep and reproducible serum proteomic profiling (13), while untargeted metabolomics provides broad coverage of metabolic perturbations. Beyond layer-specific analyses, latent variable-based computational approaches, such as Data Integration Analysis for Biomarker discovery using Latent cOmponents (DIABLO), enable the identification of co-varying molecular features across omics layers at the sample level and are particularly suited for exploratory studies aimed at uncovering systemic molecular patterns rather than constructing predictive diagnostic models (14). Accordingly, the objective of this study was not to establish a diagnostic classifier, but to explore whether circulating proteomic and metabolomic alterations in OEM show coordinated organization across biological layers.

Despite increasing application of proteomic and metabolomic approaches in endometriosis research, whether immune-related proteomic alterations and metabolic changes are coordinated at the systemic level in OEM remains unclear. To address this question, we performed label-free DIA-based serum proteomic profiling and untargeted metabolomic analysis in women with OEM and healthy controls. By integrating differential analysis with cross-omics approaches, we aimed to explore pathway-level correspondence between circulating immune-related proteins and metabolic alterations and to identify molecular features that may reflect systemic biological changes associated with OEM.

## 2 Materials and methods

### 2.1 Study design, patient recruitment, and serum sample collection

This was a single-center, case–control exploratory study of systemic molecular alterations associated with OEM. Women with OEM and healthy controls without evidence of endometriosis were consecutively recruited at Shenzhen Maternity & Child Healthcare Hospital between April 2024 and October 2024. OEM was diagnosed by laparoscopic surgery with postoperative histopathological confirmation. All included OEM patients had unilateral or bilateral ovarian endometriomas, with at least one lesion larger than 4 cm, and were scheduled for surgical management. Healthy controls were recruited from women undergoing routine health examinations or benign gynecological procedures, with no clinical, imaging, or surgical evidence of endometriosis.

The discovery cohort included six OEM patients and six age-matched healthy controls. After an overnight fast of at least 12 h, peripheral venous blood samples were collected using vacuum blood collection tubes. Samples were allowed to clot at room temperature and subsequently centrifuged to obtain serum, which was aliquoted and stored at −80 °C until analysis. Sample processing and data acquisition were performed in a randomized order, and all laboratory procedures were conducted with operators blinded to group allocation. An independent cohort was additionally recruited for ELISA-based validation, as described in Section 2.6.

Clinical information, including age, BMI, menstrual cycle phase at blood collection, CA125 level, hormonal therapy history, smoking status, reproductive history, rASRM stage, and lesion laterality, was collected from medical records where available. Menstrual cycle phase was estimated based on the interval from the first day of the last menstrual period. Available clinical characteristics are summarized in Supplementary Table S1.

The study was approved by the Ethics Committee of Shenzhen Maternity and Child Healthcare Hospital, Guangdong, China (SFYLS[2024]060), and all patients provided written informed consent.

### 2.2 Serum proteomic analysis by 4D DIA mass spectrometry

Serum proteomic profiling was performed using four-dimensional data-independent acquisition (4D-DIA) mass spectrometry. Proteins were extracted from each serum sample using a lysis buffer containing urea and protease inhibitors, followed by sonication and centrifugation at 15,000 × g for 10 min. Protein concentrations were determined using a bicinchoninic acid (BCA) assay. Proteins were reduced with dithiothreitol and alkylated with iodoacetamide under standard conditions, followed by overnight tryptic digestion using a filter-aided sample preparation (FASP) protocol. The resulting peptides were desalted using C18 solid-phase extraction cartridges and dried under vacuum. Peptide separation was performed on a nano-ultra-high-performance liquid chromatography system coupled to a timsTOF Pro 2 mass spectrometer (Bruker Daltonics, Germany), operated in parallel accumulation-serial fragmentation (PASEF) DIA mode. Peptides were separated on a 25 cm C18 analytical column at a flow rate of 300 nL/min using a linear gradient of acetonitrile. Mass spectrometry data were acquired over an m/z range of 100–1700 with an ion mobility range of 1/K0 = 0.75–1.30 V·s/cm^2^. Variable isolation windows and a stepped collision energy scheme were applied to optimize peptide coverage. Raw DIA data were processed using Spectronaut (version 18.0) against the UniProt human protein database. Trypsin was specified as the digestion enzyme with up to two missed cleavages allowed. Carbamidomethylation of cysteine was set as a fixed modification, while oxidation of methionine and N-terminal acetylation were set as variable modifications. The false discovery rate (FDR) was controlled at < 1% at both the peptide and protein levels.

Protein quantification was based on summed peptide intensities. Proteins detected in fewer than 20% of samples were excluded, and missing values were imputed using 50% of the minimum detected value for each protein. Data were median-normalized prior to downstream analysis. Proteomic data quality was assessed at the platform level using the vendor-generated report, including peptide identification metrics and global quantitative performance measures such as protein intensity distribution, hierarchical clustering, sample correlation, principal component analysis, and repeatability assessment. Because all discovery samples were processed within the same analytical workflow, no additional batch correction was applied. Differential protein abundance between groups was assessed using the Wilcoxon rank-sum test. For exploratory prioritization rather than formal biomarker declaration, proteins were selected using nominal *p* values together with effect size criteria (|log2FC| > 0.5 and *p* < 0.05), while Benjamini-Hochberg adjusted p values were additionally calculated and provided for transparency. Functional enrichment analyses were performed using Gene Ontology and KEGG databases via clusterProfiler (v4.10.1).

### 2.3 Untargeted metabolomic profiling of serum samples

Untargeted metabolomic profiling of serum samples was conducted using ultra-performance liquid chromatography coupled with tandem mass spectrometry (UPLC–MS/MS). Serum samples were prepared following standard metabolite extraction procedures and analyzed on a Q Exactive mass spectrometer (Thermo Fisher Scientific) coupled to an UPLC system. Data acquisition was performed in both positive and negative electrospray ionization modes. Chromatographic separation was achieved using a reversed-phase C18 column with water and acetonitrile, each containing 0.1% formic acid, as mobile phases under a gradient elution program. Quality control (QC) samples were included during LC–MS/MS acquisition to monitor analytical stability. Raw LC–MS data were processed using XCMS for peak detection, alignment, and feature extraction. After normalization, features with a coefficient of variation greater than 50% in QC samples were excluded from downstream differential analysis. The remaining data matrix was normalized using probabilistic quotient normalization. Global metabolic variation and sample clustering were evaluated using PCA, including QC samples for quality assessment. All discovery samples were analyzed within a single analytical batch, and no additional batch correction was applied.

Differential metabolite features were prioritized using a combination of univariate and multivariate criteria, including Welch’s t test, fold change, and variable importance in projection derived from partial least squares discriminant analysis. For exploratory prioritization of altered metabolite features, the screening criteria were Welch’s t-test *p* < 0.05, fold change > 1.5 or < 1/1.5, and VIP > 1, while Benjamini-Hochberg adjusted *p* values were additionally calculated for reference. Metabolite annotation was performed by matching accurate mass and MS/MS fragmentation spectra against an in-house spectral library and public databases, including MassBank, HMDB and LIPID MAPS database. For downstream biological interpretation, only metabolite features with high-confidence putative annotations supported by MS/MS spectral matching were retained. These annotated metabolites corresponded to MSI level 2 identifications. Features annotated only by precursor mass matching were not treated as confidently identified metabolites in downstream classification or pathway-level interpretation. Pathway enrichment analysis was conducted using the MetaboAnalyst platform^1^ based on the annotated metabolites.

### 2.4 Cross-omics integrative and pathway analysis

To explore relationships between molecular alterations across proteomic and metabolomic layers, cross-omics integration analyses were performed. Pairwise associations between prioritized proteins and metabolite features were evaluated using Spearman rank correlation analysis in R (version 4.3.3). Correlation coefficients were calculated across all samples, and protein-metabolite pairs with an absolute correlation coefficient greater than 0.5 were retained for downstream analysis, primarily for integrative visualization and descriptive assessment of cross-omics covariation. These correlations were used to characterize patterns of covariation rather than to infer direct biochemical interactions. Because correlations were calculated across all samples in a small case–control cohort, the observed covariance patterns may partly reflect group structure and should therefore be interpreted cautiously. Spearman correlation *p* values were adjusted using the Benjamini-Hochberg method. To evaluate the potential influence of case–control structure, within-group correlation analyses were performed separately in OEM and control samples as a sensitivity analysis. For comparability with the pooled analysis, the same prioritized feature subset was used, restricted to proteins and metabolites available in the matched proteomic and metabolomic matrices.

To identify biological pathways represented at both the proteomic and metabolomic levels, pathway-level integration was conducted using the Joint Pathway Analysis module implemented in the MetaboAnalyst platform. Prioritized proteins and annotated metabolites were mapped to KEGG pathways. Pathway significance was evaluated based on combined enrichment statistics using nominal *p* values, and pathway impact scores were calculated using topology-based measures provided by MetaboAnalyst.

To visually summarize the correspondence between proteomic and metabolomic alterations within shared pathways, chord diagrams were generated using the circlize R package (version 0.4.16). Chord plots were used to depict relationships between proteins, metabolites, and KEGG pathways mapped across the two omics layers, facilitating visualization of shared pathway associations.

### 2.5 Multi-omics integration using DIABLO

Multi-omics analysis was performed using DIABLO implemented in the mixOmics R package (version 6.26.0).

Proteomic and metabolomic data matrices were first filtered to remove features with missing values in more than 30% of samples. Remaining missing values were imputed using the median value of each feature. The data were subsequently log_2_-transformed and standardized to zero mean and unit variance prior to integration. Proteomic and metabolomic datasets were treated as separate data blocks, with sample group (OEM vs. CON) specified as the outcome variable. Model tuning was conducted using five-fold cross-validation repeated ten times to identify the optimal number of variables retained in each block. The tuning process aimed to minimize the balanced error rate under a full design matrix. Based on this procedure, the first latent component showed the clearest separation of sample scores between groups and was selected for downstream interpretation.

DIABLO was applied in this study as an exploratory integrative framework to identify co-varying molecular features across omics layers rather than to construct or evaluate a predictive classification model. Features with the highest loadings on the selected latent component were extracted, and protein-metabolite associations among these features were summarized based on correlation structure. The resulting interaction networks were visualized using Cytoscape (version 3.10.3).

Model robustness was further assessed using repeated M-fold cross-validation, permutation testing, and feature selection stability analysis. Cross-validated performance was summarized by balanced error rate (BER). For permutation testing, class labels were randomly shuffled and the resulting BER distribution was compared with the observed BER. Feature stability was evaluated by repeated stratified subsampling, in which one sample from each group was left out at each iteration and the selection frequency of each retained feature was calculated.

### 2.6 Enzyme-linked immunosorbent assay (ELISA)

Serum levels of DIABLO-selected platelet-associated proteins were quantified using ELISA in an independent validation cohort. An independent cohort comprising 10 women with OEM and 9 healthy controls was recruited for ELISA analysis. Serum samples used for ELISA were newly collected and independent from those included in the proteomic and metabolomic discovery analyses. All samples were stored at −80 °C and thawed on ice immediately prior to assay. Following thawing, samples were centrifuged to remove particulate debris and diluted according to the manufacturers’ recommendations.

Commercially available human ELISA kits were used to quantify platelet factor 4 (PF4; Cloud-Clone Corp., SEA172Hu), pro-platelet basic protein (PPBP/CXCL7; Cloud-Clone Corp., SEA370Hu), platelet-derived growth factor subunit A (PDGFA; Cloud-Clone Corp., SEA582Hu), and multimerin-1 (MMRN1; Cloud-Clone Corp., SEC622Hu). All assays were performed according to the manufacturers’ protocols. Serum samples and standards were measured in duplicate, and absorbance was recorded at 450 nm using a microplate reader. Protein concentrations were calculated based on standard curves generated by serial dilution of the supplied standards.

For statistical analysis, duplicate technical measurements were averaged for each sample before group comparison. Normality and variance homogeneity were assessed using the Shapiro–Wilk and Levene’s tests, respectively. Between-group comparisons were performed using Welch’s t-test, and mean differences with 95% confidence intervals were calculated. Mann–Whitney U tests were additionally performed as sensitivity analyses. Statistical analyses were conducted in R, and *p* < 0.05 was considered statistically significant.

### 2.7 Statistical considerations

Given the exploratory design and limited sample size, statistical analyses were primarily used to prioritize molecular features for integrative interpretation rather than to establish definitive biomarkers. For differential protein and metabolite analyses, nominal *p* values were used together with effect size and multivariate criteria for feature selection, while Benjamini-Hochberg adjusted *p* values were additionally calculated for transparency. Cross-omics correlation and multivariate integration analyses were interpreted as descriptive and hypothesis-generating. All findings should therefore be considered exploratory and require further validation in larger cohorts.

## 3 Results

### 3.1 Overview of the study workflow

**Figure 1:**
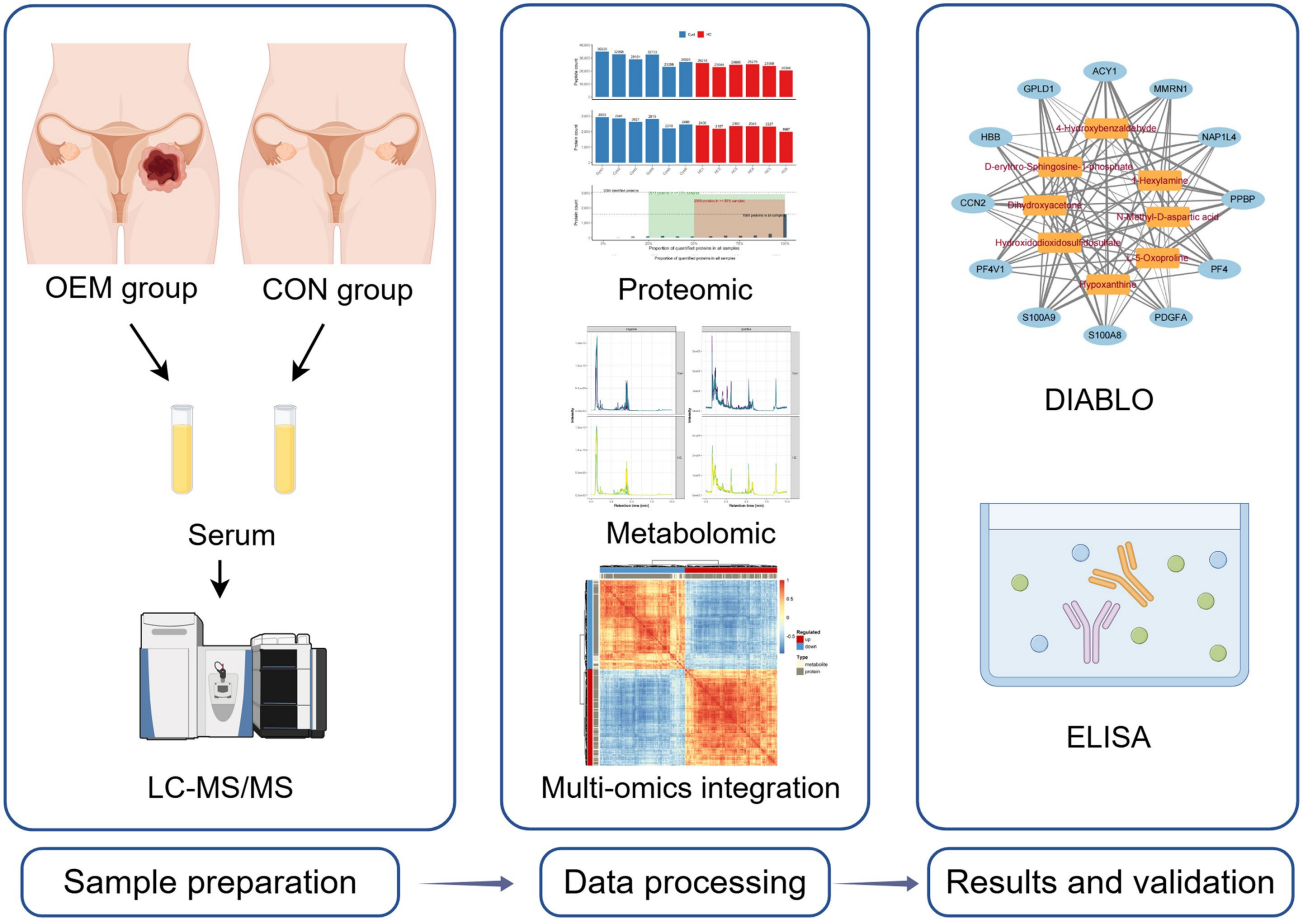
Overview of the study design and multi-omics analysis workflow.

The overall study design and analytical workflow are summarized in Figure 1. Serum samples from women with OEM and healthy controls were subjected to parallel serum proteomic and untargeted metabolomic profiling. Differential analyses were performed separately for each omics layer to identify molecular features associated with OEM. Proteomic and metabolomic results were subsequently integrated using correlation analysis, pathway-level integration, and multi-omics modeling. Selected platelet-associated proteins identified from integrative analyses were further evaluated by ELISA in an independent validation cohort. Available clinical characteristics of participants in the discovery and ELISA validation cohorts are summarized in Supplementary Table S1.

### 3.2 Proteomics alterations in serum samples from women with OEM

**Figure 2:**
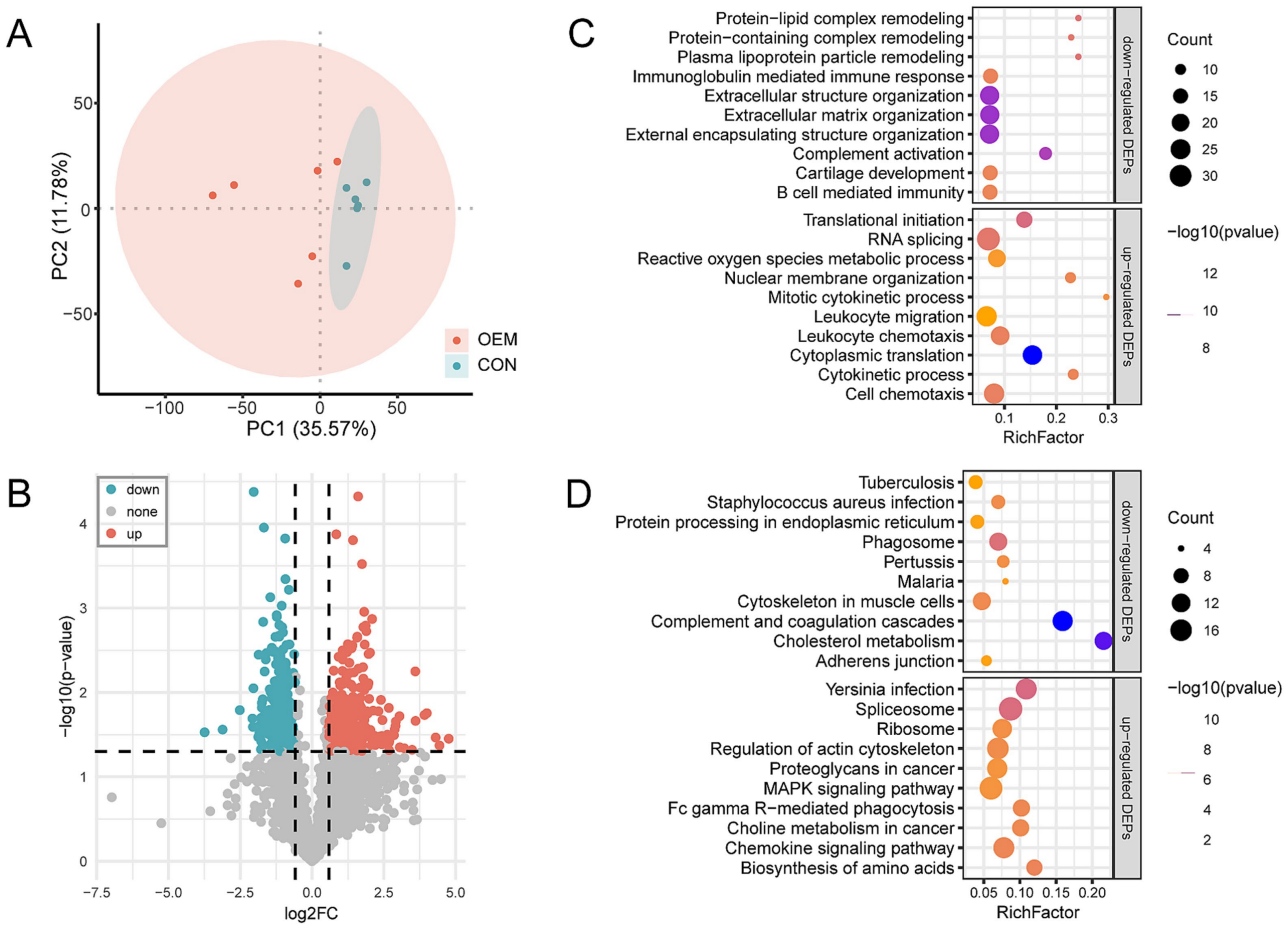
Serum proteomic alterations and functional enrichment analysis in OEM. **(A)** PCA of the serum proteomic dataset showing separation between OEM and CON samples. **(B)** Volcano plot of proteins prioritized as altered under the exploratory criteria used in this study. The dashed lines indicate the exploratory screening thresholds of |log2FC| > 0.5 and nominal *p* < 0.05. Adjusted *p* values are reported in the corresponding supplementary tables. **(C)** GO enrichment analysis of prioritized altered proteins, highlighting immune chemotaxis, RNA splicing, ROS metabolism, and extracellular matrix organization. **(D)** KEGG pathways showing up-regulated immune signaling (Chemokine, FcγR, MAPK) and down-regulated complement, coagulation, and metabolic processes.

After quality control, a total of 36,875 peptides corresponding to 3,054 proteins were identified across all serum samples. Among these, 2,563 proteins with less than 50% missing values across samples were retained for downstream quantitative analysis. PCA of the serum proteomic data showed separation between OEM patients and healthy controls along the first principal component, whereas separation along subsequent components was less pronounced (Figure 2A).

Using nominal *p* values together with effect size criteria for exploratory prioritization, differential analysis identified 658 proteins with altered abundance between the two groups, including 433 with higher abundance and 225 proteins with lower abundance in OEM serum (Figure 2B; Supplementary Table S2). Benjamini-Hochberg adjusted *p* values derived from the Wilcoxon rank-sum test are provided in Supplementary Table S2. Proteins with higher abundance in OEM serum were predominantly annotated as immune-related or extracellular proteins, whereas proteins with lower abundance were more frequently associated with complement-related and metabolic processes.

Gene Ontology enrichment analysis of proteins with higher abundance in OEM serum revealed enrichment in terms related to leukocyte chemotaxis and migration, immunoglobulin-mediated immune responses, extracellular matrix organization, and reactive oxygen species metabolic processes (Figure 2C; Supplementary Table S3). In contrast, proteins with lower abundance showed enrichment primarily in complement activation and a limited number of structural and cell cycle-related terms.

Consistent with these functional annotations, KEGG pathway analysis showed that proteins with higher abundance were enriched in chemokine signaling, Fc gamma R-mediated phagocytosis, MAPK signaling, and cytoskeleton regulation pathways, whereas proteins with lower abundance were mainly associated with complement and coagulation cascades, cholesterol metabolism, and protein processing in the endoplasmic reticulum (Figure 2D; Supplementary Table S4).

### 3.3 Metabolomic alterations in serum samples from women with OEM

**Figure 3:**
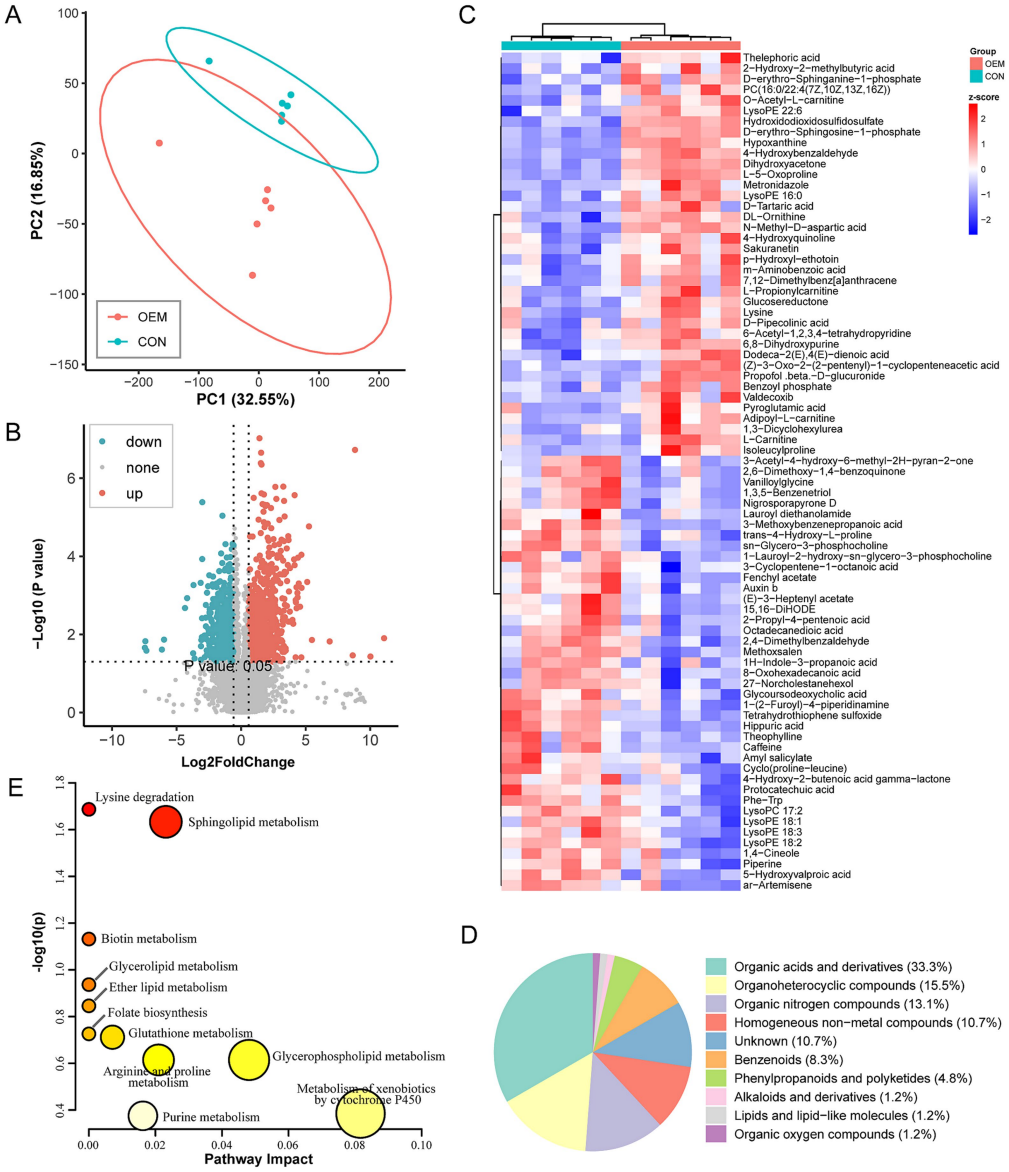
Serum metabolomic alterations and functional enrichment analysis in OEM. **(A)** PCA of the serum metabolomic dataset showing separation between OEM and CON samples. **(B)** Volcano plot of metabolite features prioritized as altered under the exploratory screening criteria. The dashed lines indicate the exploratory screening thresholds corresponding to fold change > 1.5 or < 1/1.5 and nominal *p* < 0.05. Adjusted *p* values are reported in the corresponding supplementary tables. **(C)** Heatmap of 83 high-confidence annotated altered metabolite features used for downstream interpretation. **(D)** Superclass classification of the 83 annotated altered metabolites. **(E)** KEGG pathway analysis of MSI level 2 annotated metabolites, highlighting exploratory pathway enrichment related to lipid metabolism, amino acid catabolism, and redox homeostasis.

Untargeted serum metabolomic profiling was performed using LC–MS/MS in both positive and negative ionization modes. After data processing and quality control, 10,844 features in positive mode and 8,251 features in negative mode were retained for downstream analysis. PCA showed separation between OEM and control samples, while QC samples clustered tightly, supporting the overall analytical stability of the metabolomic dataset (Figure 3A; Supplementary Figure S1).

Using Welch’s t test together with fold change and VIP criteria for exploratory prioritization, differential analysis identified 1,812 metabolite features with altered abundance between OEM and control samples (Figure 3B; Supplementary Table S5). Benjamini-Hochberg adjusted *p* values are provided in Supplementary Table S5.

To facilitate biological interpretation, we focused on 83 altered metabolite features with high-confidence putative annotations based on MS/MS spectral matching. These annotated metabolites correspond to MSI level 2 identifications and were used for downstream classification and pathway analyses. The 83 annotated metabolites were mainly classified as lipids and lipid-like molecules, organoheterocyclic compounds, organic acids and derivatives, organic nitrogen compounds, benzenoids, and phenylpropanoids and polyketides (Figures 3C,D).

KEGG pathway enrichment analysis based on these 83 MSI level 2 annotated metabolites suggested representation of pathways related to lipid metabolism, amino acid metabolism, and redox-associated processes, including sphingolipid metabolism, glycerolipid metabolism, glycerophospholipid metabolism, glutathione metabolism, and metabolism of xenobiotics by cytochrome P450 (Figure 3E). Because these metabolites were putatively annotated at MSI level 2 rather than confirmed using authentic standards, the pathway results should be interpreted as exploratory and annotation-dependent.

### 3.4 Cross-omics covariation and pathway-level integration of proteomic and metabolomic alterations

**Figure 4:**
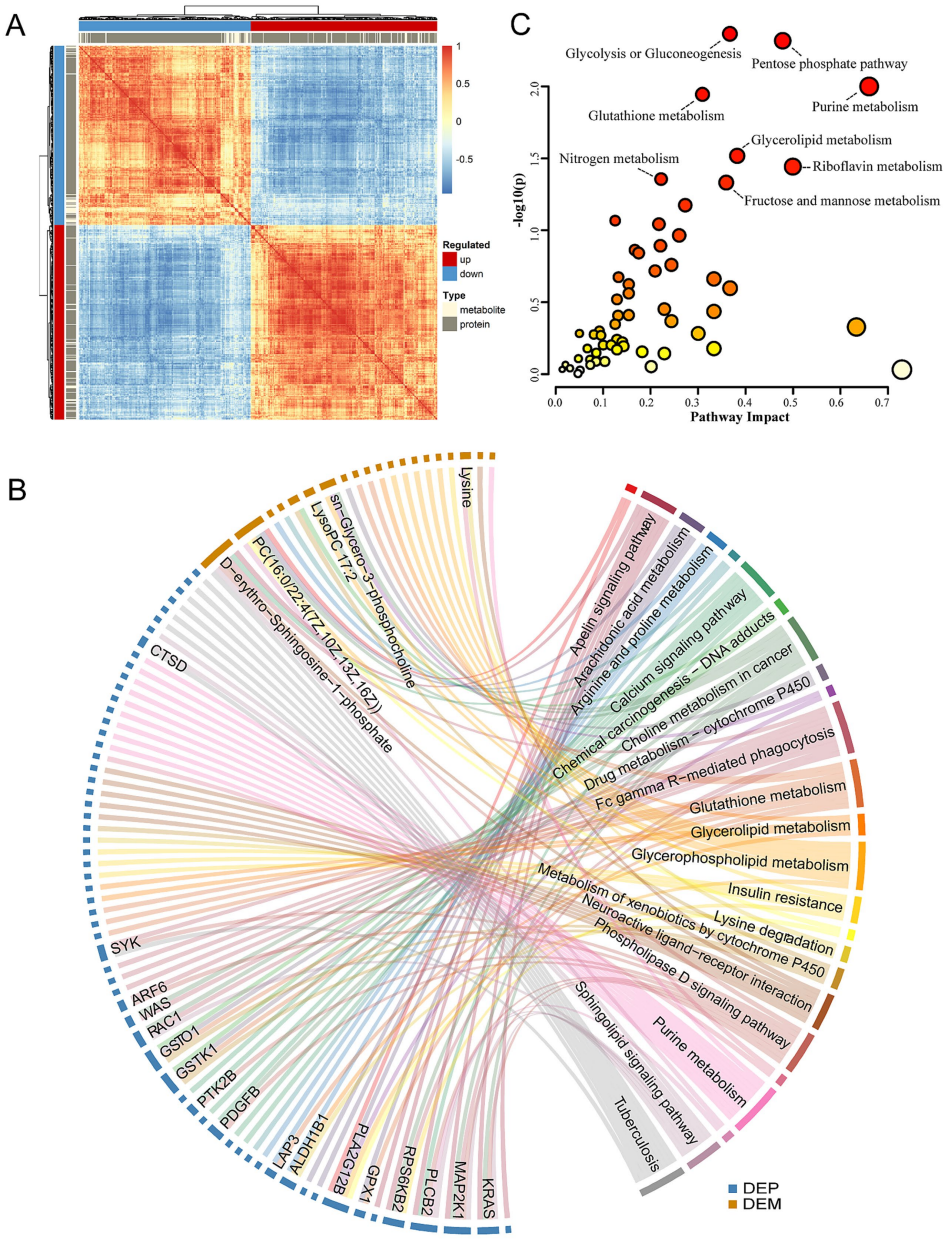
**(A)** Cross-omics covariation and pathway-level integration of proteomic and metabolomic alterations in OEM. **(B)** Correlation heatmap showing descriptive protein-metabolite covariation patterns across samples. **(C)** Chord diagram illustrating shared KEGG pathway associations mapped from prioritized proteins and annotated metabolites. Only representative enriched pathways and contributing molecules are shown. Joint pathway analysis integrating proteomic and metabolomic datasets, highlighting nominally significant pathways represented across both omics layers.

To explore relationships between proteomic and metabolomic alterations, cross-omics integration analyses were performed using prioritized proteins and metabolite features. Spearman correlation analysis was used to evaluate pairwise protein-metabolite associations across samples. Protein-metabolite pairs with absolute correlation coefficients greater than 0.5 were retained for descriptive visualization of cross-omics covariation patterns (Figure 4A), and correlation *p* values were further adjusted using the Benjamini-Hochberg method. Given the limited sample size and the possibility that correlation estimates may be influenced by group structure, these associations were interpreted descriptively rather than as evidence of direct biochemical interactions. To assess the influence of case–control structure, pooled correlations were compared with within-group correlations calculated separately in OEM and control samples. In the pooled analysis, 23,074 of 39,390 protein-metabolite pairs showed |rho| > 0.5, and 5,879 pairs remained significant after Benjamini-Hochberg correction. In contrast, substantially fewer FDR-significant pairs were observed within groups, with 163 pairs in OEM samples and 135 pairs in controls. Pooled correlations were only partially concordant with within-group correlations (Spearman correlation of rho values: 0.426 for OEM and 0.231 for controls; Supplementary Figure S2 and Supplementary Table S6). These findings further support interpreting the correlation results as descriptive cross-omics covariation rather than direct molecular interactions.

Prioritized proteins and metabolites with MSI level 2 annotations were mapped to KEGG pathways, and shared pathway associations were visualized using a chord diagram (Figure 4B). Several pathways contained both protein and metabolite components, including purine metabolism, glycolysis and gluconeogenesis, the pentose phosphate pathway, glycerolipid metabolism, and glutathione metabolism.

Joint pathway analysis identified eight pathways with nominal significance (raw *p* < 0.05). Among these, purine metabolism showed the highest pathway impact score, followed by glycolysis and gluconeogenesis, the pentose phosphate pathway, glutathione metabolism, and glycerolipid metabolism (Figure 4C; Supplementary Table S7). Together, these analyses suggest exploratory, annotation-dependent pathway-level correspondence between proteomic and metabolomic alterations in OEM, particularly involving energy metabolism, lipid remodeling, and redox-associated pathways.

### 3.5 Exploratory multi-omics integration using DIABLO

**Figure 5:**
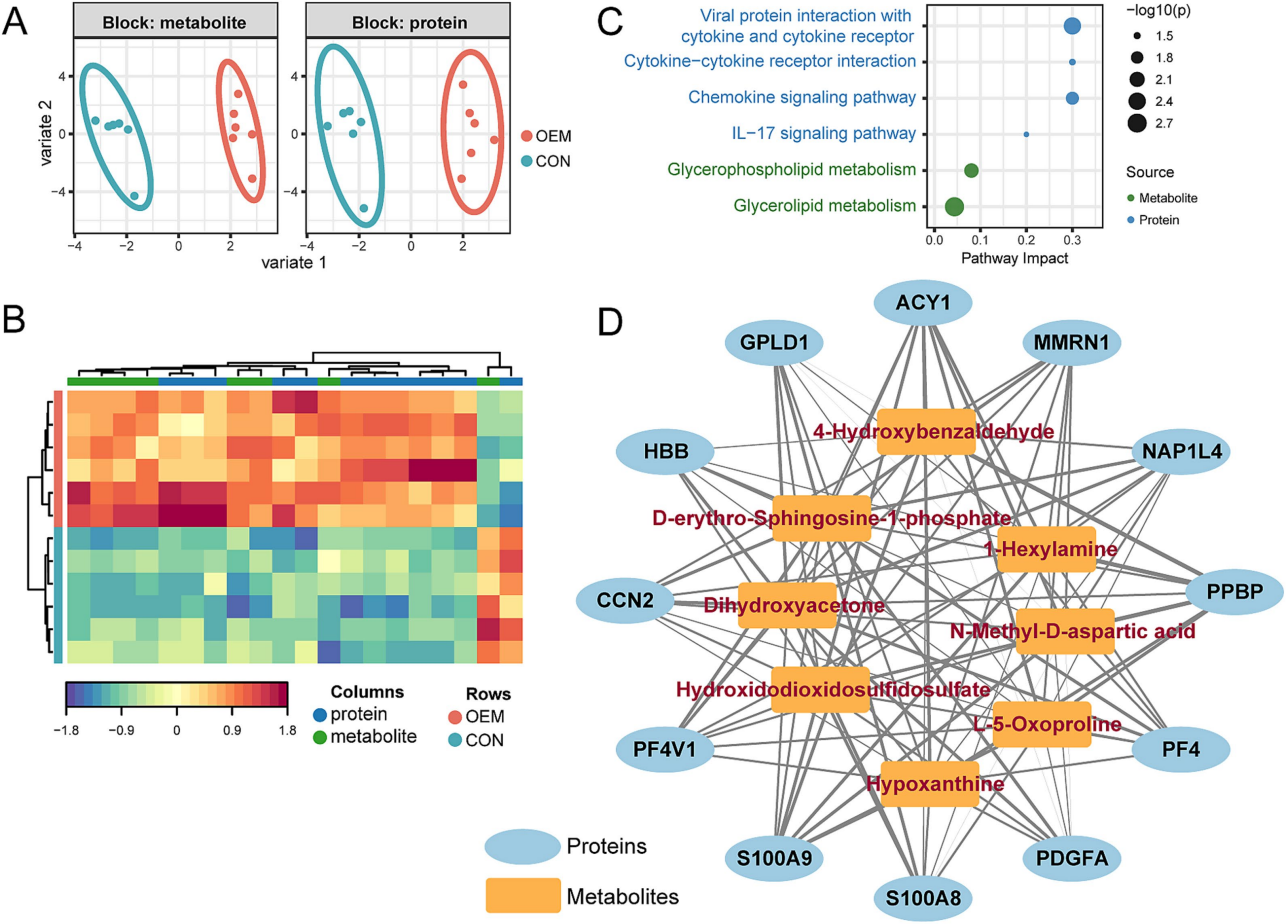
Exploratory DIABLO-based identification of co-varying multi-omics features. **(A)** DIABLO sample score plots showing the distribution of OEM and CON groups along the first latent component in the exploratory multi-omic model. **(B)** Heatmap of 12 proteins and 8 metabolites selected on the first latent component. **(C)** Functional enrichment of DIABLO-selected proteins and metabolites. **(D)** Correlation-based network visualization of protein-metabolite associations among DIABLO-selected features.

While correlation and joint pathway analyses were used to describe pairwise covariation and pathway-level overlap between the proteomic and metabolomic datasets, DIABLO was further applied as an exploratory supervised integration approach to identify multi-omics features contributing to sample-level separation between OEM and controls. Model parameters were optimized by five-fold cross-validation repeated ten times. Based on balanced error rate, 12 proteins and 8 metabolites were selected for the first latent component. Sample scores along the first component showed partial separation between OEM and control groups (Figure 5A), suggesting that the selected latent component captured sample-level covariation across the two omics layers in this exploratory dataset.

The expression patterns of the selected features were visualized using a clustered heatmap, which showed broadly consistent within-group patterns across samples (Figure 5B). To explore their potential biological relevance, pathway enrichment was performed for the DIABLO-selected features. The protein set was mainly enriched in immune and inflammatory signaling, including cytokine-cytokine receptor interaction, chemokine signaling, viral protein interaction with cytokine and cytokine receptor, and IL-17 signaling. In contrast, the selected metabolites were predominantly related to lipid and redox metabolism, with enrichment in glycerophospholipid metabolism and glutathione metabolism (Figure 5C).

Protein-metabolite associations among the selected features were summarized using correlation-based network visualization (Figure 5D). Notably, several of the selected proteins formed a connected subnetwork and were annotated as platelet-associated proteins, including PPBP, PF4, MMRN1, and PDGFA. These DIABLO-derived features should be interpreted as exploratory cross-omics signatures rather than as a validated molecular panel. Given the limited sample size, the retained features should be interpreted as exploratory co-varying signatures rather than stable or generalizable classification markers.

Additional robustness analyses were performed to evaluate the exploratory DIABLO model. Repeated cross-validation showed low BER values for component 1, with observed BERs of 0.0267 for the protein block, 0.0333 for the metabolite block, and 0.0300 for the mean BER. Permutation testing showed that these BER values were lower than expected under random class-label assignment (permutation *p* = 0.00498 for protein, 0.00995 for metabolite, and 0.00498 for mean BER; Supplementary Figure S3A and Supplementary Table S8). Feature stability analysis showed that several component 1 features were repeatedly selected across subsampling iterations, including PPBP, PF4V1, PF4, MMRN1, L-5-oxoproline, hypoxanthine, dihydroxyacetone, and D-erythro-sphingosine-1-phosphate (Supplementary Figure S3B; Supplementary Table S9). These results support non-random sample-level separation in this dataset, while the selected features remain preliminary because of the limited cohort size.

### 3.6 Exploratory validation of platelet-associated proteins by ELISA

**Figure 6:**
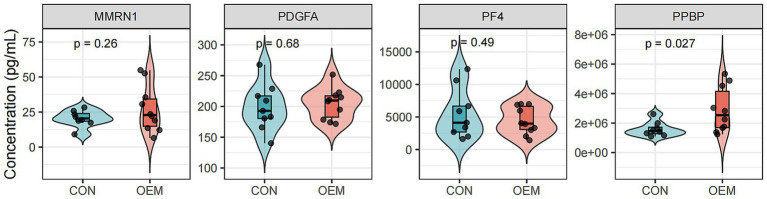
ELISA-based assessment of platelet-associated proteins in serum samples. ELISA-based measurement of PF4, PPBP, MMRN1, and PDGFA in an independent cohort, showing significant elevation of PPBP in OEM, non-significant upward trends for MMRN1 and PDGFA, and no clear group difference for PF4. ELISA *p* values were calculated using Welch’s t-test after averaging technical replicates for each sample. Mean differences, 95% confidence intervals, normality tests, variance homogeneity tests, and Mann–Whitney sensitivity analyses are summarized in Supplementary Table S10.

To experimentally assess the platelet-associated protein signal identified by the integrative analysis, ELISA was performed to quantify serum concentrations of four platelet-associated proteins, including PPBP (pro-platelet basic protein, also known as CXCL7), PF4 (platelet factor 4), MMRN1 (multimerin-1), and PDGFA (platelet-derived growth factor subunit A), in an independent validation cohort comprising 10 women with OEM and 9 healthy controls. Among the four proteins examined, PPBP showed significantly higher serum levels in the OEM group compared with controls (Figure 6). In contrast, MMRN1 and PDGFA showed increases in the same direction but did not reach statistical significance, whereas PF4 did not show a clear difference between groups.

Taken together, these results provide independent support primarily for PPBP, while also offering limited additional support for the platelet-associated signal identified in the exploratory proteomic analysis. The absence of statistical significance for the remaining proteins suggests that these candidates should still be regarded as preliminary findings requiring further validation in larger cohorts. Therefore, the ELISA results should be interpreted as partial support for a platelet-associated circulating signal rather than evidence of generalized platelet activation.

## 4 Discussion

### 4.1 Overview of the main findings

In this study, we performed an exploratory integrative analysis of serum proteomic and metabolomic profiles in women with OEM. By combining layer-specific differential analyses with cross-omics integration, we aimed to assess whether systemic molecular alterations associated with OEM exhibit coordinated organization across immune- and metabolism-related pathways. Rather than proposing a single diagnostic marker, our results suggest that OEM is accompanied by a constellation of circulating molecular changes that are detectable at the pathway level. This observation is consistent with the view that systemic immune and metabolic alterations coexist in OEM beyond localized pelvic lesions (1, 3), while also highlighting the importance of cautious interpretation given the limited cohort size.

### 4.2 Complementary proteomic and metabolomic alterations in OEM

A notable observation in this study is the limited overlap between pathways enriched in the proteomic and metabolomic datasets. Serum proteomic alterations were predominantly associated with immune-related and extracellular processes, whereas metabolomic changes mainly involved lipid metabolism, amino acid turnover, and redox regulation. We interpret this functional divergence as reflecting the distinct biological layers captured by circulating proteins and metabolites, rather than a lack of coherence between datasets. Proteins in serum are more likely to represent immune activation and intercellular signaling, whereas metabolites may capture downstream biochemical adaptations to chronic inflammatory and oxidative stress conditions. Similar functional divergence across omics layers has been reported in previous multi-omics studies of inflammatory and metabolic diseases (15, 16), indicating that limited overlap at the individual molecule level is biologically plausible and does not preclude functional convergence across omics layers.

### 4.3 Cross-omics integration

In this context, integrative analysis was informative because it enabled assessment of cross-omics relationships at the level of covariation and pathway correspondence rather than direct molecular overlap. Correlation analysis, joint pathway analysis, and DIABLO modeling consistently highlighted processes related to carbohydrate metabolism, nucleotide metabolism, lipid remodeling, and antioxidant defense, including purine metabolism, glycolysis and gluconeogenesis, the pentose phosphate pathway, glutathione metabolism, and glycerolipid metabolism. These results suggest that immune-related proteomic alterations and metabolite changes associated with redox balance and lipid metabolism may co-occur within a broader systemic context in OEM, consistent with recent literature suggesting that endometriosis involves concurrent immune-metabolic disturbance rather than a single-layer molecular abnormality (17, 18).

The three integration approaches used here served distinct purposes. Correlation analysis described pairwise protein-metabolite covariation, joint pathway analysis summarized pathways represented across both omics layers, and DIABLO identified protein and metabolite features that co-varied at the sample level and contributed to group separation in an exploratory multi-omics model. Therefore, these analyses should not be interpreted as evidence of direct biochemical interactions, stable regulatory networks, or validated classification markers. This distinction is particularly important because pooled cross-omics correlations may partly reflect case–control structure, and DIABLO-derived features remain sensitive to sample size and model overfitting. Accordingly, the integrated findings are best regarded as exploratory molecular associations that generate hypotheses for future validation.

### 4.4 Platelet-associated signatures

Among the DIABLO-selected protein features, several were annotated as platelet-associated proteins, including PPBP, PF4, MMRN1, and PDGFA. This observation suggests a possible platelet-related circulating signal in OEM, but it does not establish platelet activation as a central disease mechanism or indicate a direct mechanistic connection between platelet-associated proteins and the metabolic pathways highlighted by joint pathway analysis. Rather, these proteins should be interpreted as exploratory platelet-associated or vascular/inflammatory circulating features within the broader immune-metabolic profile observed in OEM.

This interpretation is consistent with prior serum and tissue proteomic studies reporting immune, inflammatory, adhesion, complement, and coagulation-related alterations in endometriosis, although specific candidate proteins vary across studies and sample types (4, 19). Direct evidence linking PPBP/CXCL7 to endometriosis remains limited, and PPBP should not be regarded as an established biomarker at this stage. Nevertheless, PPBP/CXCL7 is a platelet-derived CXC chemokine involved in leukocyte recruitment and inflammatory signaling, providing biological plausibility for its elevation in an inflammatory disease context. More broadly, platelets are recognized as regulators of inflammation, immune recruitment, angiogenesis, and tissue remodeling beyond hemostasis (20), and platelet activation, platelet-derived extracellular vesicles, and platelet-derived growth factor signaling have been implicated in the endometriotic microenvironment and lesion remodeling (21–23). However, similar elevations of platelet-derived chemokines in other inflammatory or immune-mediated conditions support their interpretation as components of systemic immune networks rather than disease-specific platelet markers (24, 25).

ELISA-based follow-up provided the strongest independent support for PPBP, whereas MMRN1 and PDGFA showed directionally concordant but non-significant increases and PF4 showed no clear group difference. Thus, the platelet-associated interpretation remains preliminary and is mainly supported by PPBP rather than by the full protein set. Platelet involvement should therefore be regarded as one possible explanation for the observed circulating protein pattern, while inflammatory, endothelial, vascular remodeling, and extracellular matrix-associated origins remain plausible alternative or complementary interpretations. The present study does not provide mechanistic validation, and the observed elevation of PPBP does not establish a causal role in OEM progression.

### 4.5 Clinical implications

The present findings may inform future biomarker-oriented and mechanistic studies, but they are not sufficient for immediate clinical translation. Serum multi-omics may help prioritize circulating molecular features that reflect systemic aspects of OEM biology and may complement tissue-based studies of the disease microenvironment. Among the candidates identified here, PPBP warrants further evaluation in larger independent cohorts because it was supported by both integrative proteomic analysis and ELISA validation; however, its diagnostic or prognostic utility remains unknown, and the current data do not support its use as a clinical biomarker. Future clinical application of serum multi-omics signatures will require larger multicenter cohorts, standardized sample handling, comparison with other gynecological and inflammatory conditions, and integration of clinical variables such as lesion size, disease stage, pain phenotype, infertility status, menstrual phase, and treatment history.

### 4.6 Limitations, implications, and future directions

Several limitations of this study should be acknowledged. First, this was a single-center study with a small discovery cohort, which limited statistical power and increased the risk of false-positive findings. Although Benjamini-Hochberg adjusted *p* values were calculated and robustness analyses were added for DIABLO, feature prioritization still relied partly on nominal significance combined with effect size and multivariate criteria, therefore, the results should be regarded as hypothesis-generating than definitive biomarker selection. Cross-omics correlations were also estimated across pooled case–control samples and may therefore be partly influenced by group structure. In addition, metabolite interpretation was based on MSI level 2 putative identifications rather than reference-standard-confirmed level 1 identifications, and conclusions regarding purine metabolism, glycolysis, glutathione metabolism, glycerolipid metabolism, and related pathways require validation by targeted metabolomics using authentic standards.

Experimental and clinical validation also remained limited. ELISA provided independent support mainly for PPBP, whereas MMRN1 and PDGFA should only directionally concordant but non-significant increases and PF4 showed no clear group difference. Thus, the broader platelet-associated protein set remains incompletely validated. The small cohort size and incomplete clinical information limited our ability to adjust for potential confounders such as BMI, menstrual cycle phase, CA125 level, reproductive history, smoking status, hormonal exposure, inflammatory conditions, and lesion characteristics. Finally, this study did not include mechanistic experiments; the observed elevation of PPBP does not establish a causal role in OEM progression, and the platelet-associated signal should not be interpreted as direct evidence of generalized platelet activation. Future studies should include larger, well-characterized multicenter cohorts, targeted metabolomic validation, and functional experiments to determine whether PPBP or platelet-derived factors are involved in inflammation, angiogenesis, lesion remodeling, or disease progression in OEM.

## Data Availability

The datasets presented in this study can be found in online repositories. The names of the repository/repositories and accession number(s) can be found in the article/Supplementary material.

## Footnotes

1
https://www.metaboanalyst.ca/

## Supplementary material

SUPPLEMENTARY FIGURE S1 PCA of the serum metabolomic dataset including QC samples.

SUPPLEMENTARY FIGURE S2 Pooled and within-group protein-metabolite correlations. Scatter plots compare pooled Spearman correlations with OEM-only and control-only correlations.

SUPPLEMENTARY FIGURE S3 Robustness assessment of the exploratory DIABLO model. **(A)** Permutation test for cross-validated BER. **(B)** Feature selection stability of DIABLO-selected component 1 features based on repeated stratified subsampling.
